# A Low-Cost Strain Gauge Displacement Sensor Fabricated via Shadow Mask Printing

**DOI:** 10.3390/s19214713

**Published:** 2019-10-30

**Authors:** Ying Yi, Bo Wang, Amine Bermak

**Affiliations:** Division of Information and Computing Technology, College of Science and Engineering, Hamad Bin Khalifa University, Doha 34110, Qatar; bwang@hbku.edu.qa (B.W.); abermak@hbku.edu.qa (A.B.)

**Keywords:** Flexible strain gauge displacement sensor, shadow mask, carbon ink, ink leakage

## Abstract

This work presents a cost-effective shadow mask printing approach to fabricate flexible sensors. The liquid-state sensing material can be directly brushed on a flexible substrate through a shadow mask. The ink leakage issue which often occurs in printed electronics is addressed with a custom taping scheme. A simple thermal compression bonding approach is also proposed to package the functional area of the sensor. To verify the feasibility and robustness of the proposed fabrication approach, a prototyped strain gauge displacement sensor is fabricated using carbon ink as the sensing material and a flexible polyimide (PI) film as the substrate. Once the substrate is deformed, cracks in the solidified ink layer can cause an increased resistance in the conductive path, thus achieving function of stable displacement/strain sensing. As a demonstration for displacement sensing application, this sensor is evaluated by studying its real-time resistance response under both static and dynamic mechanical loading. The fabricated sensor shows a comparable performance (with a gauge factor of ~17.6) to those fabricated using costly lithography or inkjet printing schemes, while with a significantly lower production cost.

## 1. Introduction

Strain gauge is a device that can convert an applied force to changes in resistance, capacitance, or piezoelectricity [1,2,3]. It is usually fabricated using conductive (or piezoresistive) materials such as metal thin film [4], carbon nanotube (CNT) [5], graphite nanoplatelet [6], silver nanoparticles [7], conductive polymers [8], and grapheme [9] together with a flexible or rigid substrate [10]. Recently, flexible substrate-based strain gauges become popular and have enabled various applications, such as human movement detection [5], artificial skin [8], glaucoma diagnosis [11], flexible touch panel [12], implantable sensor [13], etc. Though flexible strain sensors cannot achieve comparable sensing performance with that of silicon-based ones [10], they greatly advance in optical transparency, weight, flexibility, and fabrication cost.

Typically, patterning sensing materials on a flexible substrate involves either the transfer approach using a mask, like photolithography [14]; spray [15]; or the direct printing approach, such as soft lithography [16] or reactive inkjet printing [17]. It is known that metal deposition and lithography require complicated fabrication steps and produce toxic wastes. Therefore, they are not preferred for mass and cost-efficient manufacturing. The inkjet printing approach is capable of directly depositing functional sensing materials (in ink form) onto the substrate to form a variety of patterns, for example, a cantilever-type micro-electro-mechanical systems (MEMS) deflection sensor [18] and textile electronics [19]. However, this fabrication approach requires a dedicated inkjet printing machine. Moreover, the inkjet printing machine mandates strict physicochemical requirements on the ink, which further limits its widespread applications. For example, the ink with high solvent volatility increases the viscosity locally at the nozzles, which increases the occurrence rate of clogging [20]. 

**Figure 1:**
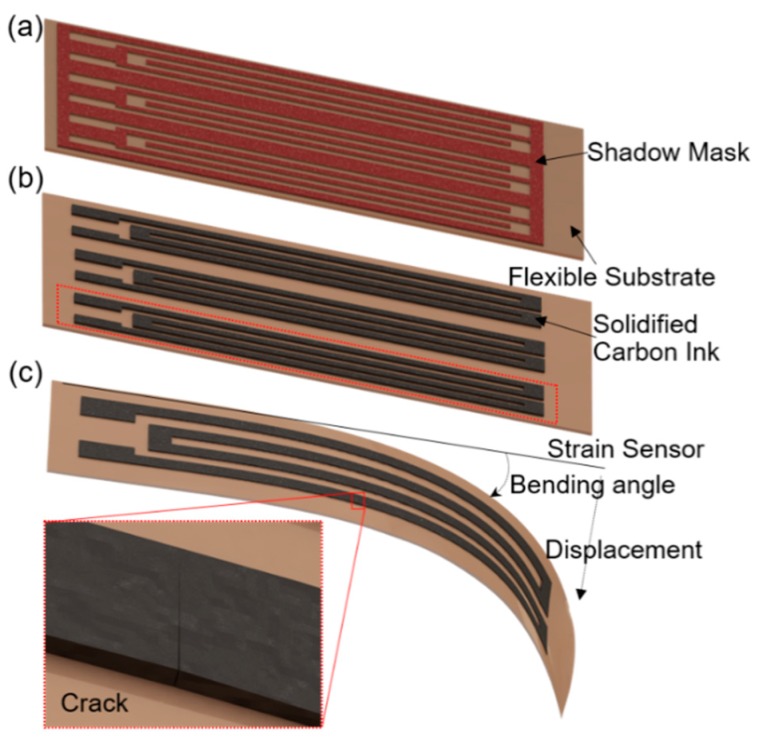
Illustrations of the fabrication and working principle of the strain sensor: (**a**) preparation of the strain sensor using a shadow mask and (**b**) printing carbon ink as the sensing material; (**c**) occurrence of crack with load applied onto the substrate.

Different from the aforementioned approaches, a new pattern process that exhibits ultra-simplicity, cost-efficiency, mass manufacturability, and compatibility with large area and high throughput processing will be presented. As shown in Figure 1, a batch of strain sensors can be produced simultaneously by using one mask layer. This mask selectively protects part of the substrate and determines the pattern of the sensor (Figure 1a). The sensing material, for example, the carbon ink used in this work, is applied on the top of the shadow mask and is solidified on the flexible substrate after the drying process (Figure 1b). An individual strain sensor sample is obtained after the substrate dicing (Figure 1c). The sensing principle is that the disconnection and reconnection of the crack fractures in the conductive links (Figure 1c) cause device resistance changes when the substrate is bent [15,21]. Obviously, this kind of cracked structure-based strain sensor fabricated using the shadow mask approach shows great potential in displacement detection, for example, structural health monitoring [22]. The remainder of this paper is organized as follows. Section 2 presents the detailed process flow of the proposed shadow mask printing approach. The working principle and characterization of the fabricated prototype strain gauge displacement sensor are introduced in Section 3, with its sensing performance presented in response to displacement detection in Section 4. Section 5 concludes this paper.

## 2. Materials and Fabrication

**Figure 2:**
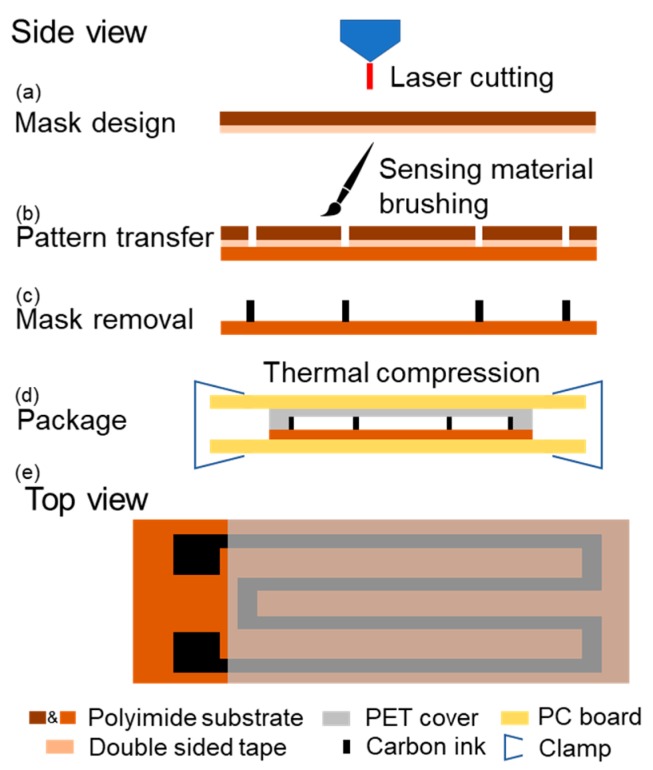
Brief illustration of the strain sensor fabrication steps using an optimized shadow mask and package approaches.

Carbon ink is a colloid-state mixture of carbon particles, polymeric stabilizers and binder and is widely used for various sensor designs [23,24]. A commercially available carbon ink is adopted in this work as the sensing material. Figure 2 shows the fabrication steps of the sensor. The fabrication starts with a mask design (using a PI layer) which determines the pattern of the solidified carbon ink on the flexible substrate. It is well known that when the mask is in direct contact with the surface of the substrate, an air gap will form between the mask and the substrate if they are not placed in vacuum conditions. As a result, the ink solvent may leak underneath the mask and mess up the desired pattern. To solve this issue, a double-sided tape (FK Double Sided Tape, thickness: ~100 μm) is adhered to the back of the mask PI film (thickness: 125 μm). A mechanical cutting machine with laser cutting function (LPKF ProtoMat D104, LPKF Laser & Electronics AG, Germany) cut through the mask layer and the tape following the desired computer-designed pattern (Figure 2a). After the laser cutting, the mask is adhered to another PI board (thickness: 125 μm) which functions as the flexible substrate of the sensor (Figure 2b). However, by back-and-forth brushing, the carbon ink can be uniformly coated to the substrate through the shadow mask (Figure 2b). A drying process that evaporates the liquid solvent in the conductive material is required to form electrically conductive paths on the substrate. In this work, the sensor is heated at 140 °C for 30 min. The mask layer including the tape is detached from the substrate after the drying process. The desired pattern of solidified carbon ink is then obtained (Figure 2c). 

**Figure 3:**
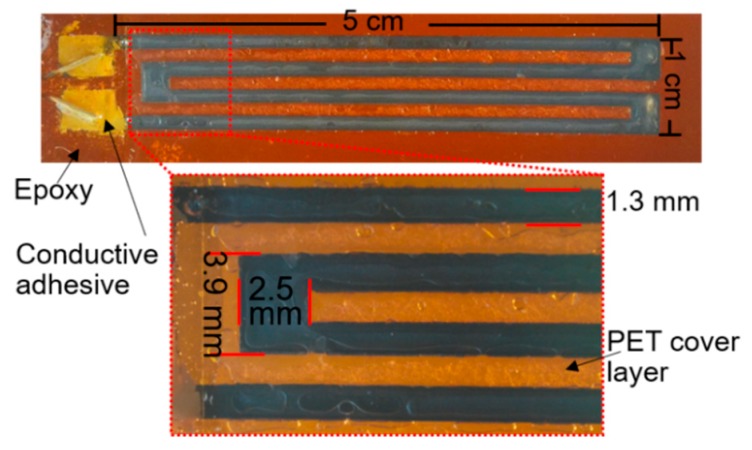
Photograph of printed strain sensor on a PI substrate (the inset shows the resulting uniform ink pattern and device dimensions).

Regarding the packaging of the sensor, we propose a simple thermal compression bonding approach that does not require complicated thermal compression instruments [25]. Firstly, a polyethylene terephthalate (PET) film is attached to the sensing area of the PI substrate (Figure 2d). Next, they are placed in between two flat rigid boards of which the edges are tightened by clamps. For example, printed circuit boards (PCB) are adopted in this work. This custom tightening setup is placed into an oven with a working temperature of 140 °C for 2 hours. The PET layer is thermally bonded with the PI substrate, covering the sensing area (Figure 2e). Following the thermal compression bonding, the two contact ends of the sensor are electrically bonded to extension wires through conductive adhesive. Finally, the two contact pads are covered by epoxy adhesive (Gorilla Epoxy, Cincinnati, OH, USA) for protection and insulation. The prototype of the fabricated strain sensor and its detailed dimensions are shown in Figure 3. A meandered shape is designed to extend the length of the conductive path. Using the proposed scheme, no ink leakage is observed during the fabrication and the resulted sensing pattern is quite uniform, as shown in Figure 3. It is worth mentioning that the sensors with different dimensions can also be fabricated following the steps above. 

## 3. Working Principle and Characterization of the Fabricated Sensor

### 3.1. Working Principle 

**Figure 4:**
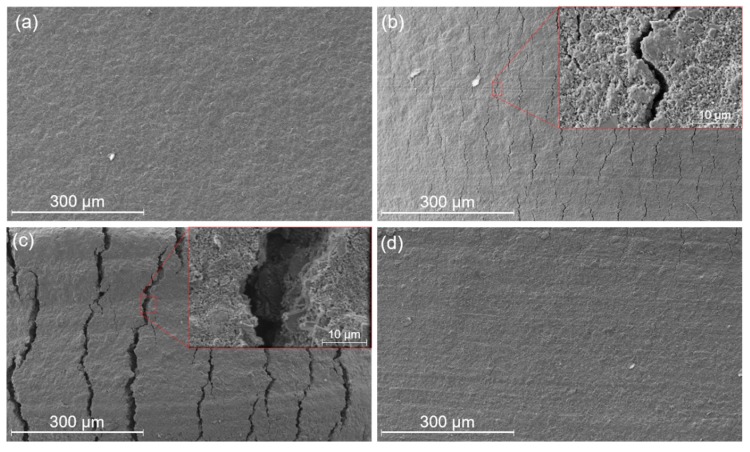
SEM images of the surface of solidified carbon ink layer on the PI substrate showing (**a**) initial state, (**b**) cracks at bending of 45°, (**c**) cracks at bending of 90°, and (**d**) restoring of cracks.

The working principle of the strain sensor is based on the change in contact resistances between the micron-scale cracked fracture surfaces. The initial surface of the solidified carbon ink is shown in Figure 4a. When the PI substrate is mechanically bent to 45°, the ink surface ruptures and cracks (~3 μm in width) emerge in the longitudinal direction, dividing the intact surface into partially disconnected regions (Figure 4b). With an increased bending angle up to 90° (maximum safe bending limit), the gap of the cracked fracture surfaces becomes larger (~20 μm in width as shown in Figure 4c), which further breaks the conductive path. As shown in Figure 4d, the PI substrate is restored to its original flat state and the fracture surfaces of the cracks are mostly reconnected after the applied strain is removed. These SEM images clearly illustrate the crack-aggregating morphology of the carbon ink layer. The increase in the width caused by the substrate deformation could result in device resistance variations. Meanwhile, without permanent mechanical deformation of the PI substrate, the disconnection and reconnection of the ink cracks are all reversible.

### 3.2. Device Characterization 

In order to evaluate the robustness of the proposed shadow mask printing approach, three sensor samples were fabricated in a single batch. The initial resistances of the sensors are 4.17 ± 0.17 kΩ at their flat conditions, corresponding to a sample-to-sample mean resistance difference of 4%. Limited by the carbon material and the substrate, the maximum operating temperature of the sensor is ~150 °C, with a temperature coefficient of the resistance presented in Figure S1. The influence of ambient temperature on the resistance of the sensor can be mitigated by calibration technologies. The Young’s modulus of the fabricated sensor can be derived from the stress-strain curve given by Figure S2. Since the prototype sensor utilizes carbon crack to achieve displacement sensing, lateral strain in the sensor would affect the cracking state and thus the sensor performance. Therefore, the sensor was characterized in two testing cases, including soft bending without lateral strain and rigid bending with strong lateral strain.

**Figure 5:**
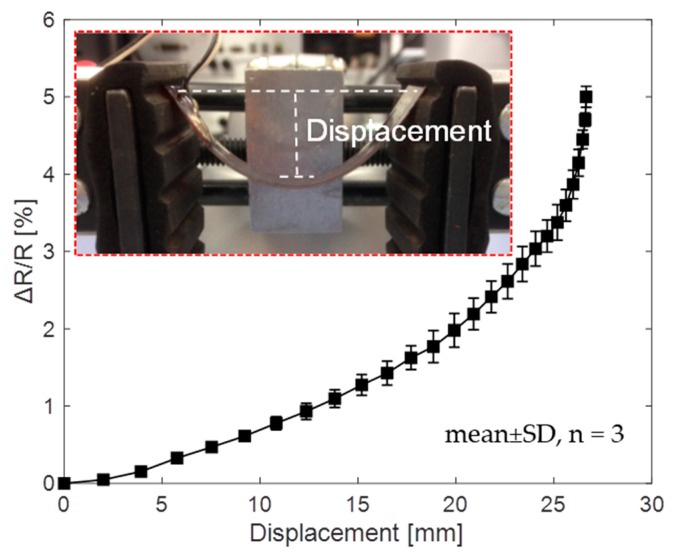
Resistance response to various displacement values when the sensor is bent.

To test the sensing performance without lateral strains, the sensor was placed on a positioning controlled mechanical fixture (Panavise Multi-Purpose Work Center, Reno, NV, USA) to achieve different deformations of the substrate, as shown in Figure 5. The normalized sensor resistance change, ΔR/R (where ΔR is the difference between the resistances at bending and relaxation states, R is the initial resistance), is shown in Figure 5. As expected, ΔR/R increased monotonically with the center displacement (or bending) of the sensor due to the enlarged cracks in the carbon ink layer (also illustrated by Figure 4). Within a safe bending limit of 27 mm (bending angle was about 90°), ΔR/R of 5% was achieved. In this test, the two ends of the sensor were not physically fixed so that the bending in the vertical direction was the main cause of the resistance variation. Under similar bending conditions, the sensor device that was fabricated using the proposed shadow mask printing approach can provide higher sensing sensitivity than the ones fabricated using lithography [26] or inkjet printing approach [27].

**Figure 6:**
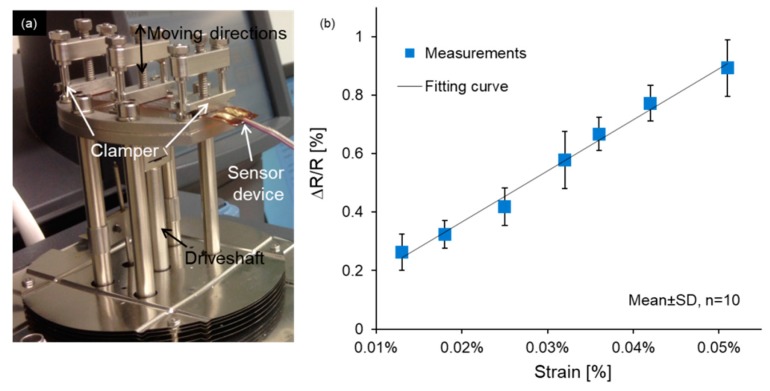
Experimental measurements use (**a**) dual cantilever mode of the DMA and (**b**) show the relationship between the ΔR/R and bending strain in the stretching process.

In order to characterize the sensing performance under strong lateral strain, the sensor device was clamped at both ends of the dynamic mechanical analyzer (DMA Q800, TA Instruments, New Castle, DE, USA) and flexed in the middle. The corresponding experimental setup was illustrated in Figure 6a. A digital multimeters (GDM-8351, GW Instek Corp., New Taipei City, Taiwan) was used to record the resistance outputs of the sensor under different levels of tension. The driveshaft moved upwards, stretching the device; the same operation was repeated 10 times for each strain value, and the blue dots of Figure 6b depicted the average ΔR/R under different strains. It is clearly seen that the sensor exhibited a linear increase trend of ΔR/R with an increasing strain (denoted as ε) in the measurement range up to 0.051% within which a maximum ΔR/R of 0.9 ± 0.1% was achieved. This result corresponds to a gauge factor [(ΔR/R)/ε] of ~17.6, which demonstrates that the fabricated sensor can provide a much higher gauge factor than the metal gauges [28] and achieve a comparable sensing performance to the textile strain sensor using polymer composites and carbon black [28,29].

**Figure 7:**
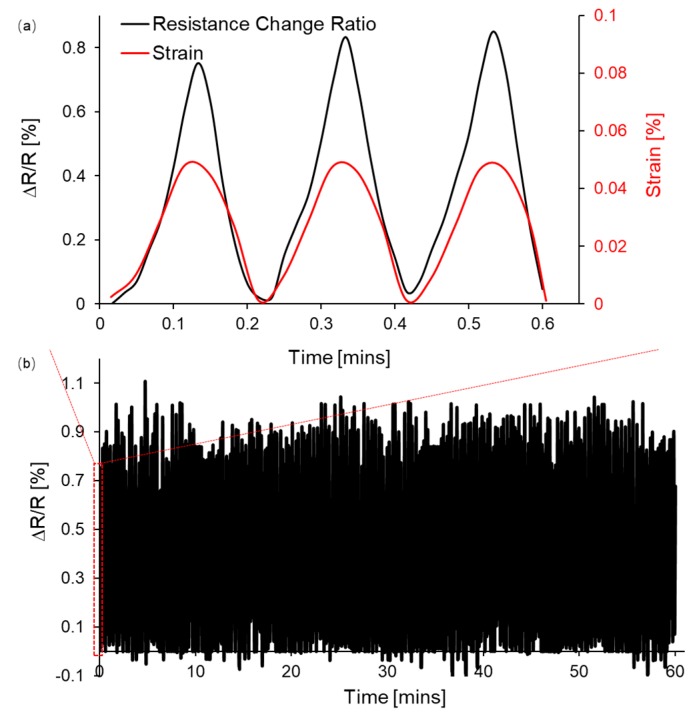
The normalized sensor resistance changes upon the strain in cyclic operations. (**a**) A magnified image of (**b**) 300 cycles of stretching and releasing.

To further investigate the robustness and repeatability of the sensor, multiple bending operations were implemented. Figure 7 shows the real-time resistance change of the sensor during 300 cycles of stretching and releasing, within each cycle the strain was increased to 0.05% for 6 s and eventually removed in another 6 s. Figure 7a gives zoom-in data showing three cycles of applied strain and the corresponding ΔR/R, which illustrates the consistency of the resistance change upon the external tension. In cyclic operations, the peak ΔR/R at ε = 0.05% is maintained at an average value of 0.9% as shown in Figure 7b, implying the reliability of the sensor. After 300 cycles of the tension test, the pattern of the sensor remained the same without any mechanical failure in the strain range of ε < 0.05%.

## 4. Application in Displacement Detection

**Figure 8:**
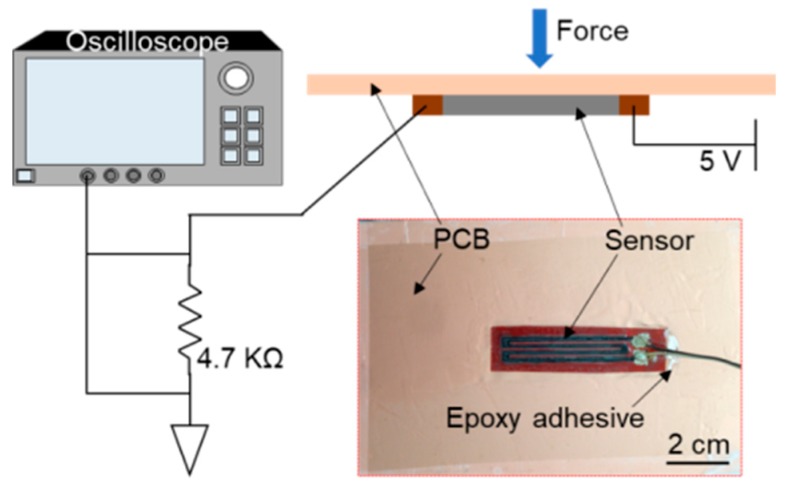
Schematic illustration of the experimental set-up and prototyped sensor device.

The experimental results above convince the sensing ability of the developed device to both soft and rigid bending conditions. As for the engineering application of the device, it can be used for detecting displacement, for example, structural health monitoring of composite bridges [22,30]. To demonstrate such potential, the sensor was attached to a copper clad laminate PCB (Paramount FR-4 059, Sunnyvale, CA, USA), in order to monitor the deformation of the board upon the external force. Figure 8 shows the deployment of the sensor on the PCB (using epoxy adhesive) and schematic illustration of the experimental setup. Once the PCB was mechanically loaded, the induced deformation would cause the sensor to bend in the vertical direction and to stretch in the horizontal direction simultaneously. A simple voltage divider circuit was used to test the transient behavior of the sensor with a 5 V supply. The voltage across the 4.7 kΩ dividing resistor was recorded by an oscilloscope (MSO 5204B, Tektronix, Beaverton, OR, USA). Different mechanical loads were applied to the center of the board, causing different levels of downward displacement. 

**Figure 9:**
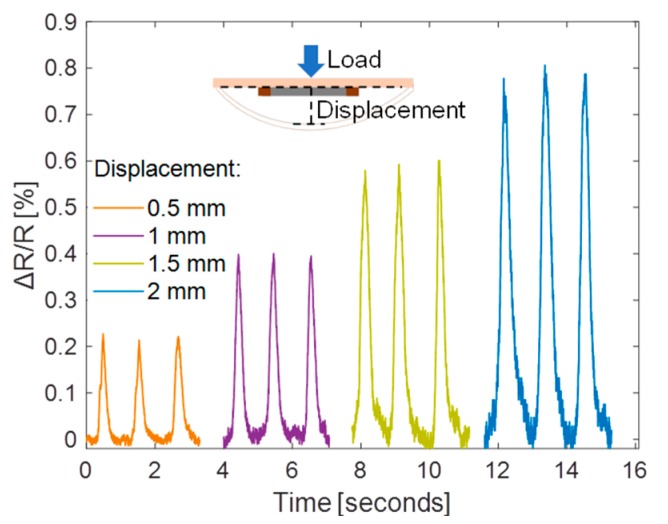
The normalized sensor resistance change at different displacement levels.

**Figure 10:**
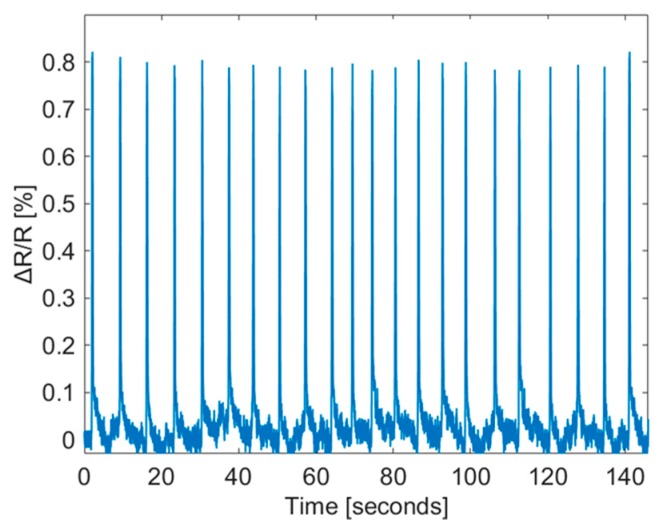
The normalized sensor resistance changes in repeated cycles of loading/unloading at displacement level of 2 mm.

Figure 9 shows the measured ∆R/R at different levels of displacement. The same operation was repeated three times for each loading test. The peak of ∆R/R increased correspondingly for the displacement being increased from 0.5 to 2 mm. The peak ∆R/R values are 0.221 ± 0.007%, 0.4 ± 0.003%, 0.6 ± 0.011%, and 0.8 ± 0.01% for a displacement of 0.5, 1, 1.5 and 2 mm, respectively. Such a variation is reasonable as the carbon cracking process is stochastic. The mechanical strain ε is 0.046% under a displacement of 2 mm [15], which corresponds to a gauge factor [(ΔR/R)/ε] of 17.4. To further verify the reliability and stability of the fabricated sensor and the fabrication method, the sensor was measured under repeated loading/unloading operations with deforming the PCB to a maximum displacement of 2 mm. Figure 10 shows that the peak ∆R/R is 0.8 ± 0.02% and the sensor can recover to its base resistance in each unloading cycle. 

**Figure 11:**
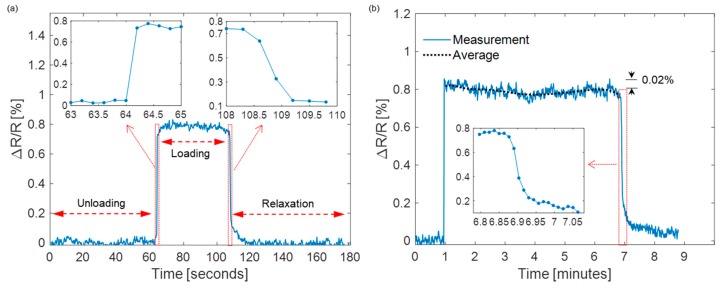
The normalized sensor resistance change under static loading conditions for (**a**) ~45 s and (**b**) ~6 min showing response to the applied load and relaxation times.

Finally, the durability of the sensor under static loading was evaluated. The displacement of 2 mm of the PCB caused by the constant applied load was maintained for about 45 s (case #1) and 6 min (case #2). In the following experiments, the sensor was repeatedly bent and released over 300 cycles until the resistance change ratio reached a stable value, such that there would be no more propagation of cracks afterwards [31]. The corresponding ∆R/R measurements are shown in Figure 11a,b, respectively. In case #1, the average ∆R/R over the entire loading period is 0.8%, which is the same as that in the cyclic operations. The insets of Figure 11a show the detailed transient responses of the sensor. When the load was applied, the response time of the sensor was about 0.2 s, after which ∆R/R gradually increased to a stable level of ~0.8%. Once the load was removed, ∆R/R decreased to 0.1% within about 1 s, followed by a slow recovering process of the carbon cracks. On the whole, the sensor can revert back to its original state within about 10 s, which implied a negligible hysteresis performance in this loading test. For case #2, an average ΔR/R of 0.81% was maintained during the beginning of the loading (<1 min) as shown in Figure 11b, while it slightly dropped to ~0.79% with time. This resistance drift is a reasonable outcome due to the stretchable nature of the substrate over a long static loading period [32]. In this case, the recovering time of the sensor was longer (>2 min) due to a longer recovering time of the board to its flat state. The measured maximum strain of the sensor is 0.15%, above which the sensor is overstretched and cannot recover. To sum up, these experimental results reveal that the sensor device shows comparable mechanical durability but higher sensitivity to those sensors with similar working principle [15,21,31], while it is patterned using the low-cost shadow mask approach.

## 5. Conclusions

In this work, a strain gauge displacement sensor fabricated using an optimized shadow mask printing approach is presented. Ink leakage issue is solved and a custom package method of the sensor is developed. The delamination of the carbon ink and polyethylene terephthalate (PET) package layers was not observed during experiments, convincing us of the robustness of the fabrication steps. Following the fabrication approach, the sensor shows an increasing ΔR/R with an increasing mechanical bending level due to the enlarging cracked fracture surfaces of carbon ink. A maximum ΔR/R of 5% is achieved at a displacement of 27 mm of the sensor when there is no lateral strain applied. Under strong lateral strain (or tension), experimental results reveal that the developed sensor can maintain its repeatable sensing characteristics (average ΔR/R = 0.9% at ε = 0.05%). As a demonstration for the displacement sensing application, the sensor that attached to the PCB exhibits similar sensing performance (e.g., ΔR/R = 0.81% at ε = 0.046%) under dynamic mechanical loading, as well as fast temporal response (0.2 s) to the applied load. Under static mechanical loading, the device shows durable sensing and excellent recovery abilities under a constant loading period up to 6 min. To summarize, the sensor using our proposed cost-efficient and simple shadow mask printing approach is reproducible, flexible, durable, robust, and sensitive to the external force (bending or tension), which suggests a great potential for displacement (or vibration) and strain detection applications. The correlated fabrication process can also be used for the development of other low-cost flexible and printable sensors. 

## Supplementary Materials

The following are available online at https://www.mdpi.com/1424-8220/19/21/4713/s1, Figure S1: Resistance of a carbon ink sensor as a function of the temperature, Figure S2: Stress-strain response shows elastic modulus.
