# Supplementary material for: A Low-Cost Strain Gauge Displacement Sensor Fabricated via Shadow Mask Printing
Source: Sensors (Basel). 2019 Oct 30;19(21):4713. doi: 10.3390/s19214713 (PMC6864542; doi:10.3390/s19214713)
Supplement: Supplementary file 1 [file sensors-19-04713-s001.docx]

Supplemental material

A Low-Cost Strain Gauge Displacement Sensor Fabricated Via Shadow Mask Printing

Ying Yi*, Bo Wang and Amine Bermak

Division of Information and Computing Technology, College of Science and Engineering, Hamad Bin Khalifa University, Education City 34110, Qatar; [bwang@hbku.edu.qa](javascript:void(0);) (B.W.); [abermak@hbku.edu.qa](javascript:void(0);) (A.B.)

***** Correspondence: yyi@hbku.edu.qa

The dynamic mechanical analyzer (DMA Q800, TA Instruments, New Castle, DE, USA) was used to characterize the physical properties of the sensor using the carbon ink, including the temperature coefficient of resistance and elasticity. The sensor was placed into the furnace of the DMA, and its resistance was measured as a function of the temperature. Figure S1 shows that the resistance is almost linearly increased with the temperature, and a temperature coefficient of the resistance (∆R/∆T) of 15.3 Ω/° was obtained within the temperature range up to ~75°. In a tension test, there is a safe range of strain in which no permanent deformation of the device occurs. As shown in Figure S2, the tensile stress was linearly increased with an increasing strain up to 0.051%, this implied that the strain ε = 0.051% was within the elastic limit. The gradient of the curve is the Young’s modulus, thus the fabricated sensor gave a Young’s modulus of ~4 GPa when ε < 0.051%.


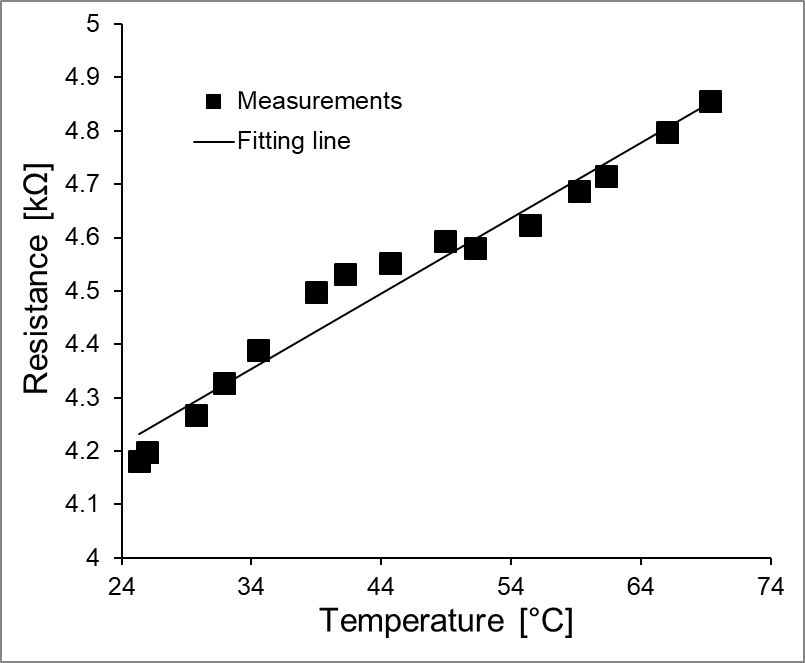


**Figure S1.** Resistance of a carbon ink sensor as a function of the temperature*.*


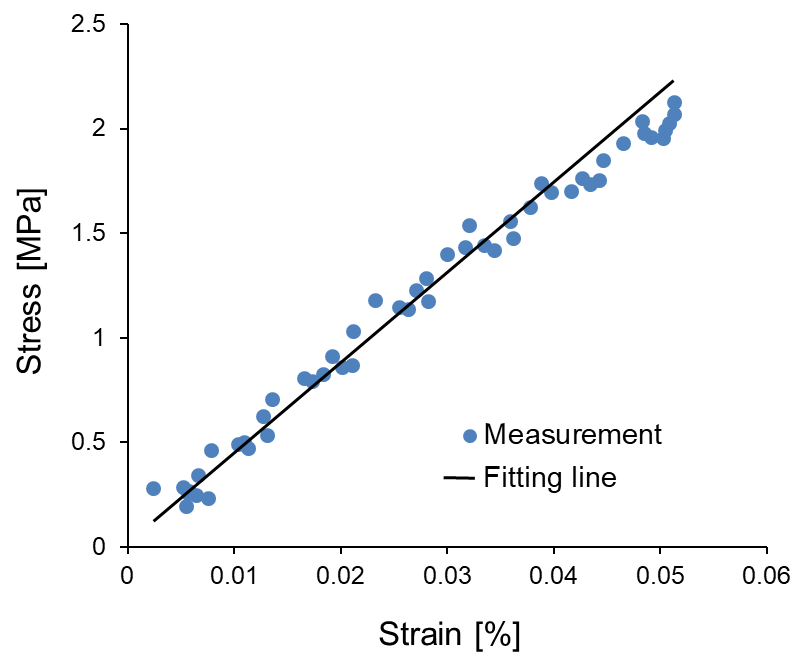


**Figure S2.** Stress-strain response shows elastic modulus*.*
